# Site‐specific and endothelial‐mediated dysfunction of the alveolar‐capillary barrier in response to lipopolysaccharides

**DOI:** 10.1111/jcmm.13421

**Published:** 2017-12-05

**Authors:** Harshavardhan Janga, Liam Cassidy, Fanlu Wang, Dietmar Spengler, Stefanie Oestern‐Fitschen, Martin F. Krause, Andreas Seekamp, Andreas Tholey, Sabine Fuchs

**Affiliations:** ^1^ Department of Trauma Surgery and Orthopedics Experimental Trauma Surgery University Medical Center Schleswig‐Holstein Kiel Germany; ^2^ Systematic Proteomics & Bioanalytics Institut für Experimentelle Medizin Christian‐Albrechts‐Universität zu Kiel Kiel Germany; ^3^ Department of Pediatrics University Medical Center Schleswig‐ Holstein Kiel Germany

**Keywords:** alveolar‐capillary barrier of the lung, lipopolysaccharides, epithelial–endothelial crosstalk, Liquid chromatography–mass spectrometry‐based proteomics

## Abstract

Infectious agents such as lipopolysaccharides (LPS) challenge the functional properties of the alveolar‐capillary barrier (ACB) in the lung. In this study, we analyse the site‐specific effects of LPS on the ACB and reveal the effects on the individual cell types and the ACB as a functional unit. Monocultures of H441 epithelial cells and co‐cultures of H441 with endothelial cells cultured on Transwells^®^ were treated with LPS from the apical or basolateral compartment. Barrier properties were analysed by the transepithelial electrical resistance (TEER), by transport assays, and immunostaining and assessment of tight junctional molecules at protein level. Furthermore, pro‐inflammatory cytokines and immune‐modulatory molecules were evaluated by ELISA and semiquantitative real‐time PCR. Liquid chromatography–mass spectrometry‐based proteomics (LS‐MS) was used to identify proteins and effector molecules secreted by endothelial cells in response to LPS. In co‐cultures treated with LPS from the basolateral compartment, we noticed a significant reduction of TEER, increased permeability and induction of pro‐inflammatory cytokines. Conversely, apical treatment did not affect the barrier. No changes were noticed in H441 monoculture upon LPS treatment. However, LPS resulted in an increased expression of pro‐inflammatory cytokines such as IL‐6 in OEC and in turn induced the reduction of TEER and an increase in SP‐A expression in H441 monoculture, and H441/OEC co‐cultures after LPS treatment from basolateral compartment. LS‐MS‐based proteomics revealed factors associated with LPS‐mediated lung injury such as ICAM‐1, VCAM‐1, Angiopoietin 2, complement factors and cathepsin S, emphasizing the role of epithelial–endothelial crosstalk in the ACB in ALI/ARDS.

## Background

Infection and associated inflammatory processes leading to acute lung injury (ALI) or its severe form, acute respiratory distress syndrome (ARDS), are major causes of morbidity and mortality in critically ill patients. Major clinical disorders that trigger ARDS comprise sepsis, pneumonia, aspiration of gastric contents and major trauma 1. The pathophysiology of ALI/ARDS is characterized by the injury and dysfunction of the pulmonary alveolar‐capillary barrier 2, 3 caused by a complex interaction of inflammatory cytokines or bacterial infection related molecules such as LPS (lipopolysaccharide) with constitutional cells in the alveolar‐capillary barrier of the lung. In addition, pulmonary microvascular endothelial cell injury and barrier dysfunction lead to the leak of protein‐rich oedema fluid and circulating neutrophils into the pulmonary interstitium and alveolar spaces 4, 5, 6. On the molecular level increased production of pro‐inflammatory cytokines 7, 8, 9, such as TNF (tumour necrosis factor), dramatically influences microtubule arrangement in the lung endothelium 10. Till date, there is no standard treatment to counteract LPS‐mediated oedema, and the complexity of the cell and molecular processes involved in the regulation of the barrier is far from being completely understood. In this context, the release of enzymes by neutrophils 11 or LPS‐induced inflammatory cytokines such as IL‐6 12, 13 is considered as major triggers for the epithelial cell damage and dysfunction in the alveolar‐capillary barrier.

In this study, we analysed the impact of LPS on the cellular interaction of endothelial cells and epithelial cells in a defined cell culture system. We analysed LPS‐mediated effects on barrier as a functional unit and the site and cell type‐specific effects of LPS in the model system. The aim of the study was to understand the molecular processes involved in the cellular crosstalk of endothelial and epithelial cells during acute phase of inflammation or infection, respectively. For this purpose, cells from the H441 alveolar‐epithelial cell line in co‐culture with microvascular endothelial cells isolated from the peripheral blood (outgrowth endothelial cells; OEC) were used as a model to assess the alveolar‐capillary barrier. In addition, the cellular response towards LPS was investigated in monocultures of the individual cell types. H441 cells share similarities with alveolar type II cells and have been shown to develop a functional epithelial barrier under suitable culture conditions in Transwell^®^ systems 14, 15. Thus, Transwells^®^ models seeded with mono‐ or co‐cultures of constitutional cells allow to analyse barrier properties and the impact of test substances in living cells under defined culture conditions.

Surprisingly, LPS had hardly any effect on H441 in the monoculture in terms of barrier properties independent from treatment from the apical or basolateral side of the barrier. Nevertheless, in co‐cultures of H441 with endothelial cells or in H441 monocultures challenged with conditioned medium from LPS‐treated OEC, the barrier properties of epithelial cells were reduced, and gene expression showed significant changes in terms of inflammation and immune‐regulatory molecules such as SP‐A. Accordingly, these results imply a strong impact of paracrine factors derived from endothelial cells on the barrier properties of epithelial cells in response to LPS. The cell culture supernatants from LPS‐treated OEC were subjected to a proteomic approach based on liquid chromatography–mass spectrometry (LC‐MS) to further identify secreted factors in microvascular endothelial cells in response to LPS to allow a better understanding of endothelial and epithelial crosstalk and the mediators involved in lung injury.

## Methods

### General cell culture protocol H441

H441 cells were cultured and maintained in RPMI 1640 medium (Gibco^®^/Life Technologies) supplemented with 10% foetal bovine serum (FBS), 1% (Pen/Strep) and 1% L‐Glutamine (Gibco^®^/Life Technologies, Darmstadt, Germany) at 37°C and 5% CO_2_. Passage numbers between 55 and 59 were used for the experiments.

### Isolation of Outgrowth endothelial cells (OECs)

OECs isolated from peripheral blood of human adults were used as a source of cells with microvascular endothelial characteristics. OECs were isolated and characterized as previously described by Fuchs *et al*. 16, 17, 18 in accordance with the approval of the local ethical advisory board and the consent of the individual donors. In brief, mononuclear cells (MNCs) were isolated from the human peripheral blood by gradient centrifugation and were cultured using endothelial growth medium‐2 (EGM‐2) (Lonza, Walkersville, MD, USA), 5% FBS (Sigma‐Aldrich), 1% penicillin/streptomycin (pen/strep) (Biochrom GmbH, Berlin, Germany), on collagen (BD Biosciences, Bedford, MA, USA) coated 24‐well plates for one week with a cell density of 5 × 10^6^ per well. After one week of culture, the adherent cells were collected by trypsinization and reseeded at a density of 5 × 10^5^ cells/well. The colonies of OEC with cobble‐stone like morphology appearing after 2–3 weeks were collected and expanded in EGM‐2 medium_._ Outgrowth microvascular endothelial cells (OECs) were cultured and maintained in EGM‐2 medium, supplemented with 5% FBS, 1% Pen/Strep.

### H441 monoculture and co‐culture experiments in the Transwell^®^ System to assess barrier properties

To measure the barrier properties, H441 cells were cultured on Transwells^®^ (polyester membrane, 0.4 mm pore size, Corning Costar, Wiesbaden, Germany) as monocultures or in co‐cultures with OEC. For mono‐ and co‐cultures experiments, H441 cells were seeded at a cell density of 1.5 × 10^5^ cells/cm^2^ on the apical surface of the Transwells^®^ containing 200 μl of RPMI 1640 medium supplemented with 10% FCS, 1% pen/strep, 1% L‐glutamine. In a co‐culture set‐up, OECs were pre‐seeded at a cell density of 5.0 × 10^4^ cells/cm^2^ on basolateral surface of the fibronectin‐coated Transwells^®^. 800 μl of EGM2 was added to the basolateral compartment of the H441 monocultures, as well as to the basolateral compartment of the co‐cultures after the adhesion of OEC. After the 3 days of cell culture, the RPMI medium in the apical compartment was supplemented with 1 μM dexamethasone (Sigma‐Aldrich Co, St Louis, USA).

Transepithelial electrical resistance (TEER) was measured using epithelial voltohmmeter (EVOM) (World Precision Instruments, Inc., Sarasota, FL USA) starting from day 3 on a daily basis, followed by medium exchange using the cell culture media as described above. TEER from empty wells was subtracted from measured values, and TEER values were expressed in ohm×cm^2^.

### Treatment with Lipopolysaccharide (LPS)

On day 5, monocultures of H441 or co‐cultures of H441 with OEC were treated with 10 or 20 μg/ml of LPS (Sigma, Deisenhofen, Germany) for 48 hrs either from apical or basolateral compartment in the Transwell^®^ culture system. In addition, OECs were treated with LPS in monocultures, being cultured on fibronectin‐coated 24‐well plates at a cell density of 5.0 × 10^4^ cells/cm^2^. On day 5, OEC treated with 10 or 20 μg/ml of LPS for 48 hrs to harvest conditioned cell culture medium and RNA. Conditioned medium from LPS‐treated OEC was used to treat the H441 monocultures from the basolateral compartment of the Transwell^®^ filter in the same way as described for the LPS treatment described before.

### Determination of paracellular permeability

H441/OEC co‐cultures on Transwells^®^ were treated with 20 μg/ml LPS as described above or left untreated as controls. Paracellular permeability for sodium fluorescein 376.27 g/mol (Sigma‐Aldrich, Steinheim, Germany) and fluorescein isothiocyanate‐dextran (FD4) 4 kD (Sigma‐Aldrich) from the apical to basolateral side was measured. Transport experiments were started by replacing the apical supernatant with 10 μg/ml of sodium fluorescein or 50 μg/ml of FD4 containing medium. In sodium fluorescein transport experiment, 50 μl of medium from the basolateral compartment was collected every 30 min. over a time period of 150 min. and replaced with an equal volume of fresh culture medium. Samples were collected on 96‐well plates, and 200 μl of 1 M NaOH was added to the samples to adjust the pH to 7.4. In FD4 transport assay, 200 μl of medium from the basolateral compartment was collected every 30 min. over a time period of 150 min. from a 96‐well plate and replaced with an equal volume of fresh culture medium. All samples were analysed in a fluorescence microplate reader (Tecan, Crailsheim, Germany) with excitation and emission wavelengths of 480 and 530 nm, respectively. Absorbance of the medium was subtracted from measured values as a blank value. During the experiment, TEER was measured to monitor the barrier integrity. The cumulative amount of sodium fluorescein or FD4 in the basolateral compartment was plotted as a function of time.

### Immuno fluorescence staining for ZO‐1

To visualize the effect of the conditioned medium derived from LPS‐treated OEC on ZO‐1 expression in H441 cells, 3.57 × 10^4^ cells/cm^2^ were cultured on Chamber Slide™ (Thermo Fisher Scientific Inc., Nunc™ Rochester, USA). The cells were then washed with phosphate‐buffered saline (PBS) (Gibco^®^/Life Technologies, UK) three times. The cells were then fixed with 4% paraformaldehyde (PFA) (Affymetrix) for 10 min. and subsequently washed three times with PBS before being permeabilized using 0.5% Triton^®^X‐100 (Sigma‐Aldrich, Taufkirchen, Germany). Cells were washed again with PBS and then blocked for 1 hr using 1% bovine serum albumin (BSA). Then cells were incubated with rabbit anti ZO‐1 antibody (Invitrogen) 1:100 diluted in 1% bovine serum albumin (BSA) in PBS for 90 min. at room temperature. After washing, samples were incubated with secondary antibody (Invitrogen‐Molecular Probes, Eugene, USA) diluted 1:1000 in 1% BSA in PBS for 1 hr. Nuclei were counterstained with Hoechst (Invitrogen‐Molecular Probes, Eugene, USA) for 5 min. and analysed using a fluorescence microscope (Zeiss, Axioskop 40, Göttingen, Germany).

### RNA, cDNA preparation and Quantitative Reverse transcription‐Polymerase chain reaction (qRT‐PCR)

To assess the gene expression of H441 cells in monocultures or in co‐cultures in response to LPS treatment, RNA was isolated from the H441 cells cultured on Transwells^®^ using RNeasy Total RNA kit (Qiagen, Hilden, Germany). Using PCR for endothelial markers, purity of RNA from epithelial cells was controlled. To assess the gene expression levels in LPS‐treated OEC monocultures; OECs were cultured on 24‐well plates with a cell density of 1 × 10^5^ cells/well, and RNA was isolated. cDNA was prepared from 1 μg of RNA using High capacity RNA to cDNA kit (Applied Biosystems, Carlsbad, USA) according to the manufacturer's protocol. The expression of different genes was determined by qRT‐PCR performed using Rotor‐Gene Q detection system (QIAGEN). For detailed information of the primers used, refer to Table 1. 12.5 μl of QuantiTect^®^ SYBR Green PCR Master Mix (Qiagen), 2.5 μl of QuantiTect^®^ primer assay (Qiagen) or 1 μl of 10 μM forward and reverse primer (Eurofins Genomics), 4 μl of 10 ng/μl cDNA and RNase free water (Qiagen) were used to adjust to a final volume of 25 μl for one reaction. All the data were further normalized using *RPL13A* as a housekeeping gene. The reaction was performed using duplicates of probes, and the reports were analysed by an approximation method, Gene expression was analysed by ΔΔct method and setting control sample value as 1 (reference value).

**Table 1:** Primer list

Gene name	Primer Assay name	Catalogue number
RPL13 A	Hs_RPL13A_1_SG	QT00089915
COL 1A1	Hs_COL1A1_1_SG	QT00037793
IL8	Hs_IL8_1_SG	QT00000322
IL1β	Hs_IL1β_1_SG	QT00021385
SFTPA2	Hs_SFTPA2_1_SG	QT00072926
SFTPC	Hs_SFTPC_1_SG	QT00000714
SFTPD	Hs_SFTPD_1_SG	QT00037219
TLR4	Hs_TLR4_2_SG	QT01670123
ZO‐1	HS_TJP1_va.1_SG	QT00077308
VCAM1	Hs‐VCAM1_1_SG	QT00018347
ICAM1	Hs_ICAM1_1_SG	QT00074900
RAGE	Hs_AGER_1_SG	QT00000119
OCLN	Hs_OCLN_va.1_SG	QT01327739
VWF	Hs_VWF_1_SG	QT00051975
CDH1 (E‐cadherin)	Hs_CDH1_1_SG	QT00080143
CTNNB1 (β‐catenin)	Hs_CTNNB1_1_SG	QT00077882

Gene name	Sequence	Annealing temperature
IL‐6	for 5′‐GCCCTCGAGCCCACCAGGAA‐3′ rev 5′‐CCCCAGGGAGAAGGCGACTG3‐3′	66°C
TNF‐α	for 5′‐AGAGCATGATCCGAGACGTGGAG‐3′ rev 5′‐AGTAGGCAGAAGAGCGTGGTGG‐3′	64°C
VEGF	for 5′‐GAGCTTCCTACAGCACAAC‐3′ rev 5′‐CAAATGCTTTCTCCGCTC‐3′	55°C
CD31	for 5′‐CCGGATCTATGACTCAGGGACCAT‐3′ rev 5′‐GGATGGCCTCTTTCTTGTCCAG‐3′	55°C

### MTS Assay

Cell viability was determined using tetrazolium compound 3‐(4,5‐dimethylthiazol‐2‐yl)‐5‐(3‐carboxymethoxyphenyl‐2‐(4‐sulfophenyl)‐2H‐tetrazolium salt (MTS) (Promega, Madison, USA). H441 or OECs were plated onto 96‐well plates with a cell density of 6.0 × 10^4^ cells/cm^2^ and were grown for 5 days before being treated with 10 or 20 μg/ml LPS or with conditioned medium from 10 or 20 μg/ml LPS‐treated OEC for 48 hrs. 20 μl of the combined MTS solution was pipetted into each well of the 96‐well plates containing 100 μl of culture medium and incubated at 37°C for 2 hrs. The formazan production was determined by measurement of the spectrometric absorbance at 560 nm. The blanks were used for background subtraction and concentrations depicted as a percentage of the untreated control (Figure S1). No significant effects on cell viability were observed in these control experiments.

### Cell surface biotinylation and protein isolation

Cells were seeded on 75‐cm^2^ flasks, with a density of 1.5 × 10^5^ per cm^2^ and cultured and treated as described before. On day 7, cell surface protein isolation was initiated using Sulfo‐NHS‐SS‐Biotin (Thermo Fisher Scientific, Pierce Rockford, IL, USA) kit. In brief, cells were washed two times with ice‐cold phosphate‐buffered saline (PBS) (Gibco, Darmstadt, Germany) followed by a 30‐min. incubation with Sulfo‐NHS‐SS‐Biotin. The non‐reacted biotin was blocked with Quenching Solution (Thermo Fisher Scientific, Pierce Rockford, IL, USA), the cells were scraped off and transferred to tubes. The cells were centrifuged, washed in TBS and lysed with buffer supplemented with protease inhibitors, transferred to 1.5 ml tubes, sonicated and incubated on ice for 30 min. Then the cell lysates were centrifuged, and supernatant was collected. The NeutrAvidin Agarose used for pulldown was added to a column, centrifuged 1 min. at 1000 × g, washed three times with wash buffer, and the cell lysate was added to the gel. The cell lysate and the gel were incubated at room temperature for 60 min., centrifuged and washed with buffer supplemented with protease inhibitors. SDS‐PAGE sample buffer (Life Technologies, Darmstadt, Germany) supplemented with 50mM DTT (Thermo Fisher Scientific, Pierce Rockford, IL, USA) was used for elution. After centrifuging, the supernatant was collected. The protein concentration was determined using the BCA (Thermo Fisher Scientific, Pierce Rockford, IL, USA) protein assay supplemented with Ionic Detergent Compatibility Reagent (Thermo Fisher Scientific, Pierce Rockford, IL, USA) at 660 nm.

Protein lysates of non‐biotinylated H441 cells were prepared in RIPA buffer. The cells were scraped off in PBS and centrifuged. The pellet was resuspended in RIPA buffer (150 mM NaCl, 1% NP‐40, 0.5% sodium deoxycholate, 0.1% SDS, 50 mM Tris, pH 8.0), supplemented with protein inhibitors (complete Mini EDTA‐free Protease Inhibitor Cocktail, Roche, Basel, Switzerland) and lysed on ice for 30 min. Samples were centrifuged at 8000 g for 20 min. The protein concentration of the supernatants was determined using BCA protein assay (Thermo Fisher Scientific, Pierce Rockford, IL, USA). Prior to SDS‐PAGE samples were heated at 70°C for 2 min. in 2 × SDS‐PAGE sample buffer.

### Western blot

Total protein extracts, respectively, biotinylated protein extracts were loaded in equal amounts per lane on Novex™ WedgeWell™ 4–20% Tris‐Glycine Gel (Invitrogen, Carlsbad, USA) and after electrophoresis using Tris‐Glycine SDS buffer (invitrogen, Carlsbad, USA) transferred to PVDF membranes (Millipore, Bedford, USA). The membranes were blocked for 1 hr at room temperature in 3% skimmed milk (BIO‐RAD, Hercules, USA). The primary antibodies Zo‐1 (rabbit), (1:200, Invitrogen, Carlsbad, USA), occludin 1:200 (rabbit), (Invitrogen, Carlsbad, USA) and phospho‐caveolin‐1 (rabbit) (1:2000, abcam, Cambridge, UK) were applied to the membranes and incubated at 4°C overnight. Beta‐actin antibody was applied as loading control. After three times wash with TBS/T buffer, the secondary antibodies goat anti‐rabbit lgG‐HRP (Santa Cruz biotechnology, Santa Cruz, USA) (1:5000 diluted) and Precision ProteinTM StrepTactin‐HRP Conjugate (BIO‐RAD, Hercules, USA) (1:10000 diluted) were applied to the membranes and incubated for 1 h at room temperature. The membranes were exposed to Pierce ECL Western Blotting Substrate (Thermo, Rockford, USA) and signals detected by High performance chemi‐luminescence films (GE healthcare, little Chalfont, UK) for further analysis using Image Studio Lite (LI‐COR, Lincoln, US).

### Quantification of different cytokines using enzyme‐linked immunosorbant assay (ELISA)

The supernatants from the apical and basolateral compartments of H441/OEC co‐cultures, H441 monocultures, and supernatants from the OEC monocultures were collected after 48 hrs of LPS treatment. The concentrations of different factors in these supernatants were determined using ELISA Duo Set^®^ IL‐6, IL‐8, MCP‐1 ELISA (R&D Systems, Wiesbaden, Germany) according to the manufacturer's instructions. The optical density of each well was measured using an automatic plate reader (Apollo, Berthold Technologies, Bad Wildbad, Germany).

### Liquid chromatography–mass spectrometry (LC‐MS)

Ten or 20 μg/ml LPS‐treated OEC conditioned medium was prepared by culturing 5 × 10^5^ OEC cells in fibronectin coated T25 flasks in 5 ml of EGM2 medium for 5 days and then treated with 10 or 20 μg/ml LPS in serum‐free EGM2 medium for 48 hrs.

Conditioned media from four OEC donors, untreated or treated with LPS, were frozen at −80°C and then lyophilized to complete dryness. After resuspension in 1 ml triethyl ammonium bicarbonate (TEAB) buffer (100 mM, pH 8.0) protein concentration was determined using Bradford protein assay.

Aliquots of approximately 12.5 μg were subjected to a short SDS‐PAGE to concentrate and desalt the samples. For this purpose, samples were dissolved in Laemmli loading buffer, heated at 95°C for 5 min. and the migrated only into the stacking gel (4%) of a standard 12% gel using a stepped voltage method (10 min. 40 V, 5 min. 80 V, 15 min. 110 V). This allowed the dye front to migrate approximately 10 mm into the gel. The gel lanes were stained with Coomassie blue, and the complete lane for each sample was excised and placed into micro test tubes. Gel lanes were sliced into small pieces (approx. 1 mm^3^), washed with MilliQ^®^ water, and ammonium bicarbonate (ABC) buffer (100 mM, pH 8) was added, and proteins were reduced with dithiothreitol (DTT) (10 mM, 56°C, 1 hr) and alkylated with iodoacetamide (IAA) (55 mM, RT in the dark, 30 min.). The samples were washed, dehydrated using acetonitrile (ACN), desiccated by vacuum evaporation and then rehydrated at 4°C for 45 min. with 100 mM ABC containing trypsin (250 ng; enzyme to protein ratio of 1:50). The samples were transferred to a shaking mixer (at 37°C), and the trypsin digestion was performed overnight. Peptides were extracted suing increasing concentrations of acetonitrile (ACN), desiccated via vacuum evaporation and resuspended in 3% ACN, 0.1% trifluoroacetic acid.

LC‐MS analysis was performed using a Dionex UltiMate 3000 RSLCnano UPLC system (Thermo, Germany) equipped with an Acclaim PepMap RSLC nano column (C18, 100 Å, 2 μm, 75 μm × 500 mm) (Thermo, Germany) coupled online to a QExative Plus mass spectrometer (Thermo, Germany) as described previously 19. The solvents used were buffer A: 0.05% formic acid (FA), buffer B: 80% ACN + 0.04% FA; the flow rate was 300 nl/min. Separation was performed over a programmed gradient: isocratic elution at 4% B for 2 min., followed by a linear gradient from 4% to 40% B over 150 min., a 8 min. linear increase to 90% B, and 9 min. isocratic at 90% B, 1 min. linear down to 4% B, followed by 15 isocratic equilibration at 4% B.

The MS analysis was performed in positive ion mode and utilizing HCD fragmentation at normalized collision energy (NCE) of 27.5. A full‐scan MS acquisition was performed (resolution 70,000, AGC target 3e6, scan range 300 to 2000 m/z in profile mode), with subsequent MS/MS (resolution 17,500, AGC target 1e5, isolation window 3.0 m/z, scan range 100 to 2000 m/z) of the top 10 most intense ions; charge exclusion was enabled for unassigned, +1 and >+8 ions. Dynamic exclusion was enabled with a 20 sec. duration, and lock mass was enabled (445.12003 m/z).

Protein identification was performed by database search using the Proteome Discoverer software package (V1.4; Thermo, Germany) applying the SequestHT algorithm; Percolator node was used for calculating the estimated false discovery rate (FDR). The raw data files were searched against a combined human protein FASTA database (20,319 entries; downloaded version 2013/07/08) and 97 common laboratory contaminants (cRAP laboratory and contact dust contaminants). Protein identification criteria were as follows: at least two high‐confidence peptides (FDR 1%) per protein, and a minimum of one unique peptide. Tolerances: precursor: 5 ppm; fragments: 0.02 Da; modifications: dynamic (oxidation Met; deamidation Asn/Gln); static: carbamidomethylation (Cys). Enzyme specificity: trypsin.

### Statistical analysis

All data if not stated otherwise are presented as mean values ± standard deviation of the mean for at least three biological replicates. The graphs and analysis were performed using the graph pad prism software V, and the statistical significance was determined by one‐way anova (*P*‐value *P < 0.05, **P < 0.01 and ***P < 0.001).

## Results

**Figure 1:**
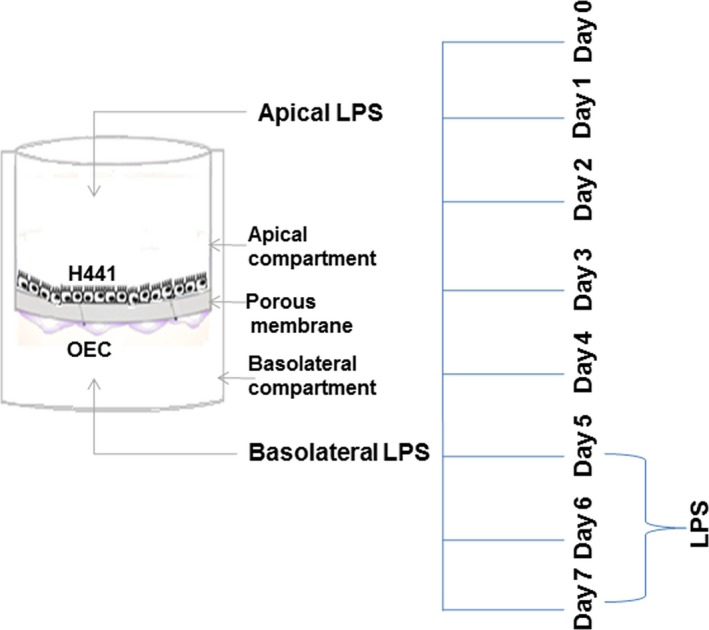
Assembly of the alveolar‐capillary barrier *in vitro* culture model and overview of the experimental set‐up.

In this study, we studied the barrier properties and cellular crosstalk of epithelial and endothelial cells in *in vitro* models of alveolar‐capillary barrier upon LPS treatment using H441 epithelial cell line in monoculture or in co‐culture with microvascular endothelial cells (outgrowth endothelial cells (OEC)). In this set‐up, the Transwell^®^ system and the formation of tight junctional complexes allow studying of site‐specific effects in the alveolar‐capillary model after exposure to model compounds. The general experimental set‐up using H441 monocultures or co‐culture with OEC in a Transwell^®^ system is depicted in Figure 1.

### Impact of outgrowth endothelial cells on the alveolar‐epithelial barrier

**Figure 2:**
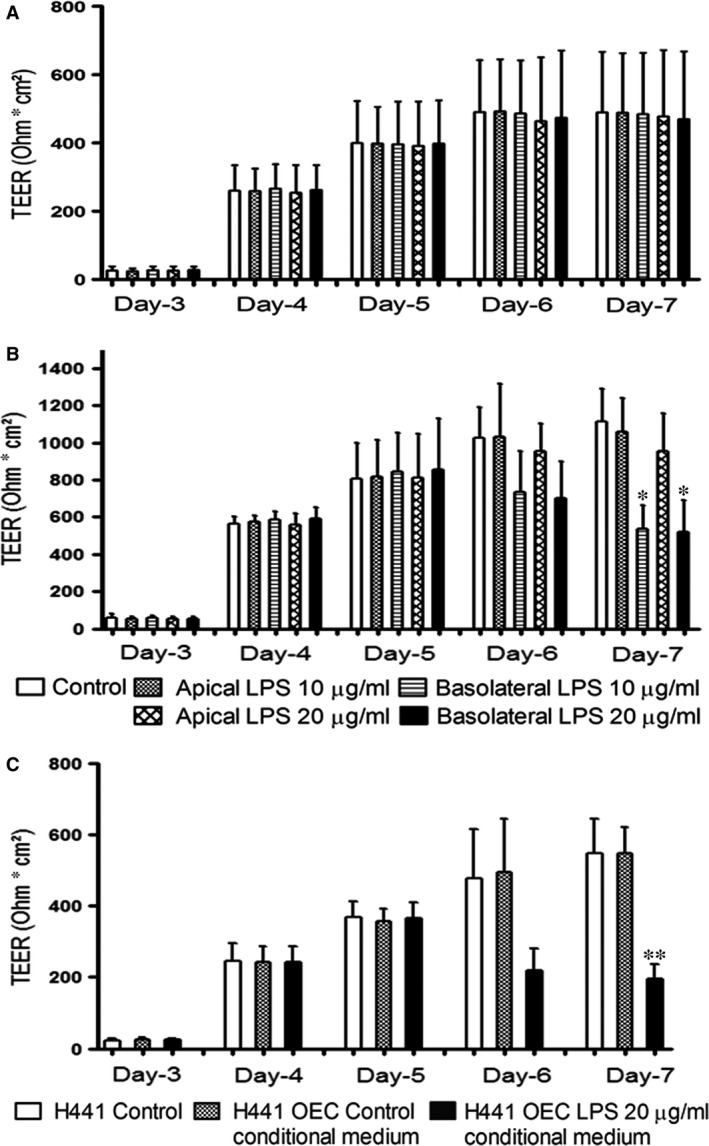
Transepithelial electrical resistance (TEER) of monocultures and co‐cultures before and after exposure to 10 or 20 μg/ml of LPS; H441 monocultures treated with 20 μg/ml of LPS‐treated OEC conditioned medium. **(A)** monoculture of H441, **(B)** co‐culture of H441 with OEC was treated 10 or 20 μg/ml of LPS from apical or basolateral compartment of transwell^®^ filters on day 5 of culture for 48 hrs. **(C)** The conditioned medium from 20 μg/ml LPS‐treated OEC was extracted and used to treat H441 monocultures on transwell^®^ filters from the basolateral compartment on day 5 of culture for 48 hrs. TEER values are depicted as Ohm cm^2^. TEER was measured from day 3, and results are shown as means + S.D. of three independent experiments (*P < 0.05, **P < 0.01).

Transepithelial electrical resistance (TEER) of monocultures and co‐cultures allows measurement of barrier properties in living cells and is shown in Figure 2 as a function of time in the culture. Maximum TEER values (untreated cells) after 7 days of cultivation yielded mean values of 1300 (±153) Ohm × cm^2^ for co‐cultures and 490 (±170) Ohm × cm^2^ in monocultures of H441 (Fig. 2). OECs in monoculture did not reveal any significant TEER values (data not shown) which are in accordance with the technical limitation to measure the barrier properties of endothelial cells with an EVOM system, but they are able to support the barrier function of epithelial cells and specifically H441 cells (Fig. 2). Accordingly using the EVOM mainly epithelial contribution to the barrier can be measured in the co‐culture, whereas the direct impact of endothelial cells on TEER is not measurable by an EVOM system.

### Site‐specific effects of LPS on the barrier of H441 monocultures and H441/OEC co‐cultures

LPS was used as model substance for bacterial endotoxins to investigate the response of the alveolar barrier towards infection. The TEER of monocultures of H441 cell line and co‐cultures of H441 with OECs was analysed over 7 days; while treatment with 10 or 20 μg/ml LPS either from the apical or basolateral compartment of Transwell^®^ filters was carried out from day 5 to day 7. When the H441 monocultures were exposed to 10 or 20 μg/ml of LPS from the apical or basolateral compartment of Transwell^®^ filters for 48 h no significant changes in TEER were observed (Fig. 2A). Even in the H441/OEC co‐cultures, no changes in the TEER occurred when treated with 10 or 20 μg/ml LPS from the apical compartment (Fig. 2B). Upon treatment with LPS from the basolateral compartment of the co‐cultures, we noticed a significant reduction in TEER for both tested LPS concentrations (Fig. 2B) suggesting an influence of soluble factors secreted by OEC on the epithelial barrier.

Thus, we evaluated in the next step the effect of soluble factors on H441 cells using conditioned medium from OEC treated with 20 μg/ml LPS. H441 monocultures were cultured for 5 days, and TEER was measured from day 3 onwards. Significant reduction in TEER (Fig. 2C) was observed when conditioned medium from LPS‐treated OEC was added to H441 monocultures from basolateral compartment of the Transwell^®^ filter.

### Immunofluorescent labelling of ZO‐1 proteins

**Figure 3:**
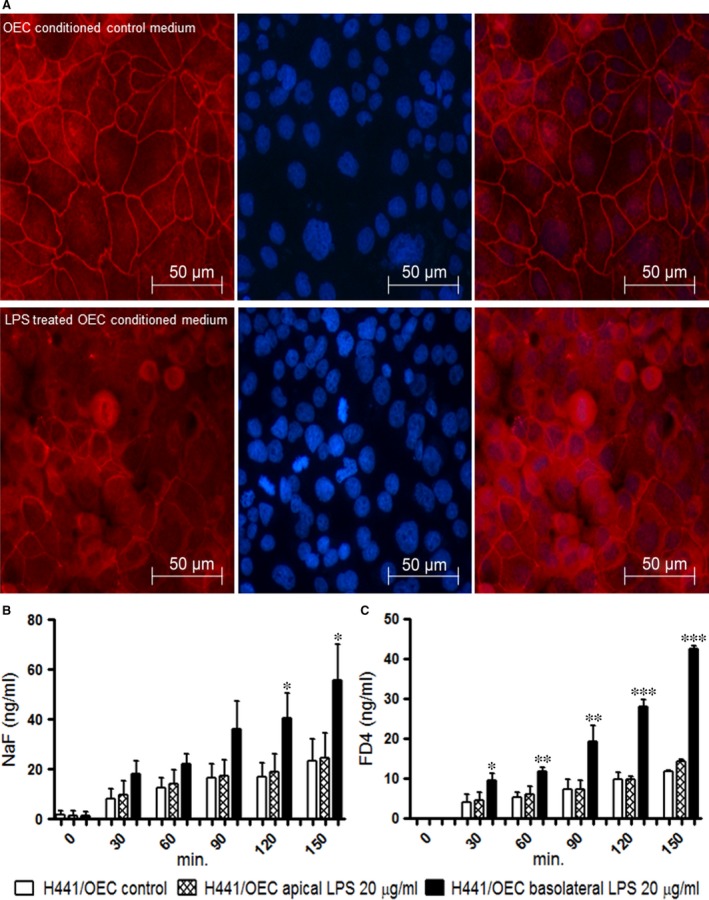
**(A, B)** ZO‐1 expression in H441 monocultures treated with OEC conditioned medium **(A)** H441 cells treated with conditioned OEC control, **(B)** H441 cells treated with conditioned medium from OEC (10 μg/ml of LPS). **(C, D)** Transport of sodium fluorescein and FD4 across cell bilayers of H441 co‐cultures with OEC after 48 hrs treatment with 20 μg/ml of LPS. **(C)** Paracellular transport of sodium fluorescein in co‐cultures from apical to basolateral compartment after exposure to 20 μg/ml of LPS either from apical or basolateral compartment **(D)** Paracellular transport of FD4 in co‐cultures from apical to basolateral compartment after exposure to 20 μg/ml of LPS either from apical or basolateral compartment. Results are shown as means + S.D. of three independent experiments (*P < 0.05, **P < 0.01 and ***P < 0.001, annova).

The integrity of the tight junctional complexes within the H441 cells and the morphological appearance of H441 cells in response to the conditioned medium were assessed by staining for the tight junction protein ZO‐1 (zonula occludens protein‐1). Exposure to conditioned medium from control groups (no LPS) showed a typical staining pattern for ZO‐1 along the cellular contacts of the epithelial cell layer. In response to the treatment of H441 cells with conditioned medium from LPS group (Fig. 3), local disruptions of the cell contacts after staining for ZO‐1 were detected. These observations are in accordance with the TEER data indicating an effect of soluble factors derived from endothelial cells on the epithelial barrier.

### Paracellular permeability of mono and co‐cultures in response to LPS treatment

Infection might lead to the flooding of the alveolar air space with protein‐rich fluid; thus, we used sodium fluorescein (376.269939 g/mol) and FITC‐Dextran (FD‐4, 4 kD), two compounds of different molecular weight, to analyse the impact of LPS on the paracellular permeability and transport processes in the co‐culture system.

Increased amounts of sodium fluorescein (Fig. 3B) or FD‐4 (Fig. 3C) molecules were transported from the apical to the basolateral compartment when co‐cultures of H441 and OEC were treated with 20 μg/ml LPS from basolateral compartment (Fig. 3B;D) in comparison with untreated controls. In contrast, LPS treatment from the apical compartment had no consequence on the paracellular permeability in the co‐culture model. In addition, in response to LPS treatment from the basolateral compartment, tentative higher transport rates for sodium fluorescein (Fig. 3B) compared to FD‐4 (Fig. 3C) were observed. These findings are in accordance with the differences in the lower molecular weight of test substances and the higher transport rates for small molecules.

### Gene expression of epithelial barrier‐associated molecules and surfactant proteins in H441 epithelial cells in response to LPS

Real‐time PCR was performed to analyse the impact of LPS on junctional molecules regulating the barrier properties of epithelial cells but also on surfactant proteins involved in immune response or acting as important constitutional molecules of alveolar‐epithelial cells.

**Figure 4:**
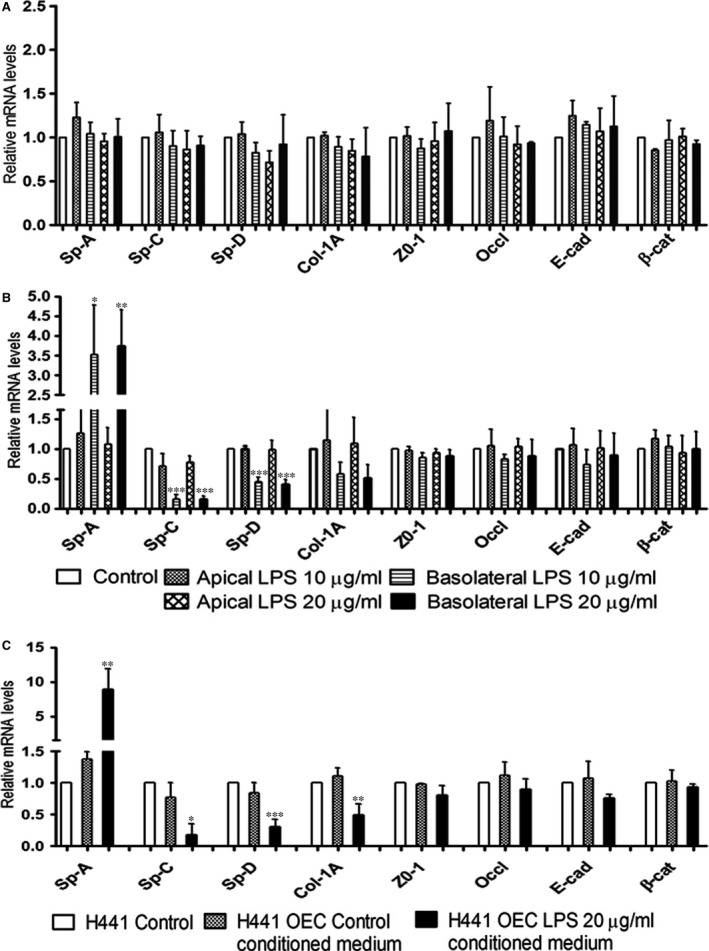
Relative quantification of gene expression of surfactant proteins, matrix components and tight junctional molecules in **(A)** monocultures, in **(B)** co‐cultures of H441 when treated with 10 or 20 μg/ml of LPS and **(C)** H441 monocultures treated with 20 μg/ml of LPS‐treated OEC conditioned medium on day 5 of culture for 48 hrs was investigated by quantitative real‐time PCR. *RPL13A* was used as an endogenous standard to normalize the data, and control was set to 1. Each bar represents the mean + S.D. of three independent experiments (*P < 0.05, **P < 0.01 and ***P < 0.001, annova).

The monocultures of H441 cells and co‐cultures of H441/OEC were treated with 10 or 20 μg/ml LPS, and gene expression in H441 epithelial cells was analysed. In H441 monocultures, no significant changes were observed for surfactant molecules (SP‐A, SP‐C, SP‐D), junctional molecules (ZO‐1, occludin, E‐cadherin, β‐catenin) and matrix molecules (col‐1A) upon LPS treatment from apical or basolateral compartment (Fig. 4A). Similar observations were found for co‐cultures of H441/OEC in response to the treatment with LPS from apical side (Fig. 4B). In contrast, after treatment of co‐cultures with LPS from the basolateral compartment, we observed a significantly up‐regulated expression of SP‐A and down‐regulation of SP‐C, SP‐D (Fig. 4B). On the other hand, no significant changes were noticed for the expression of in junctional molecules (*Z*O‐1, occludin, E‐cadherin, β‐catenin) while a slight down‐regulation of extracellular matrix molecule col‐1A was observed (Fig. 4B).

Treatment of H441 monocultures from the basolateral compartment with conditioned medium derived from OEC exposed to LPS significantly increased the expression of SP‐A and induced a down‐regulation of SP‐C, SP‐D (Fig. 4C). In addition, no significant changes were noticed in junctional molecules, but a slight down‐regulation of col‐1A molecule was noticed (Fig. 4C). Accordingly, these results were similar to the observations found for the co‐cultures after exposure to LPS from the basolateral compartment indicating a major impact of factors derived from endothelial cells on epithelial barrier properties and defence mechanisms.

### Assessment of tight junctional proteins and phosphorylated caveolin‐1 by Western blot

**Figure 5:**
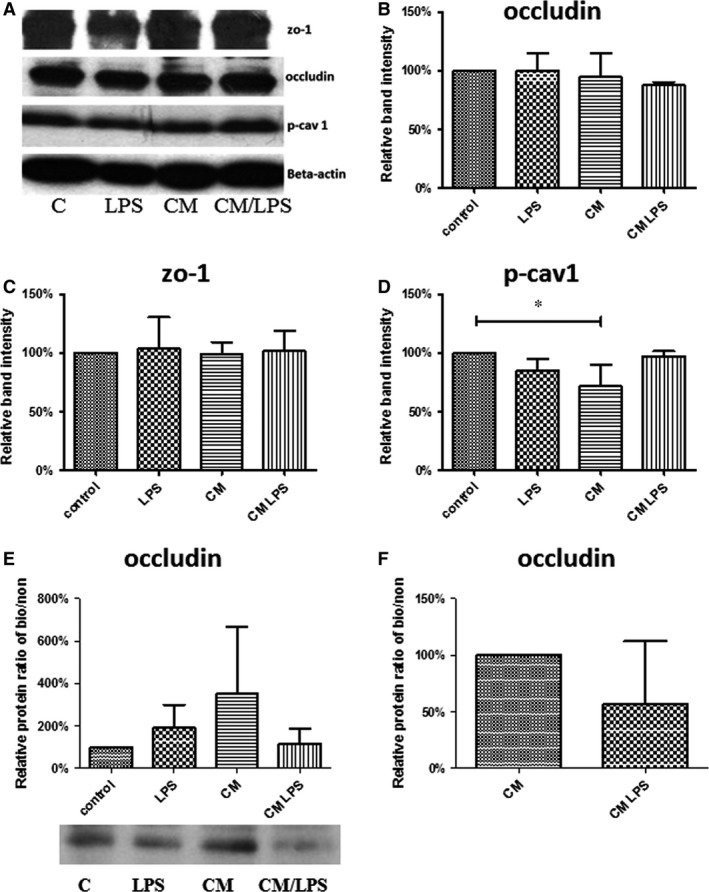
Protein analysis by Western blots for total protein depicted in **(A–D)** and after surface biotinylation depicted as ratios biotinylated/non‐biotinylated for occludin **(E,F)**. H441 cells were treated with LPS or with conditioned medium from LPS‐treated OEC, and protein lysates were generated as described before in Materials and Methods. **(A)** Representative bands at the predicted molecular weight of the corresponding proteins and beta‐actin (MW 42 kD, loading control) as indicated. **(B–F)** Quantification of band intensities. Each bar represents the mean + S.D. of three independent experiments in comparison with reference group (100%) as indicated (*P < 0.05, annova) for occludin (B, MW 65 kD), zo‐1 (C, MW 195 kD), phospho‐caveolin 1 (D, MW 20 kD), ratio biotinylated/non‐biotinylated for occludin and representative bands for occludin in biotinylated samples **(E, F)**.

In addition, we performed Western blots and quantified the tight junctional proteins ZO‐1 and occludin to analyse potential effects of LPS or OEC‐derived mediators at the protein level. Furthermore, the phosphorylated form of caveolin‐1 (p‐cav1) was investigated on the protein level. Figure 5 depicts the quantitative analysis for the total protein amount for occludin (B), ZO‐1(C) and the phosphorylated form of caveolin‐1(D). For occludin and ZO‐1, no significant changes in the total protein amounts were observed. Only p‐cav1 was slightly reduced in the conditioned medium compared to the control group. Phosphorylation of caveolin‐1 is supposed to increase in case of barrier impairment for instance in response to LPS 20, 21.

In addition, the ratio of the protein content of the tight junctional transmembrane protein occludin on the cell surface to the total occludin protein content was determined after cell surface biotinylation and streptavidin pull down. No significant differences were observed, although the ratio of surface occludin to total occludin may tentatively reflect the corresponding barrier properties as indicated by TEER and reported data for the internalization and trafficking of occludin in case of barrier impairment 22, 23.

### Site‐specific effects of LPS on the inflammatory activation in H441 monoculture or in H441/OEC co‐cultures

**Figure 6:**
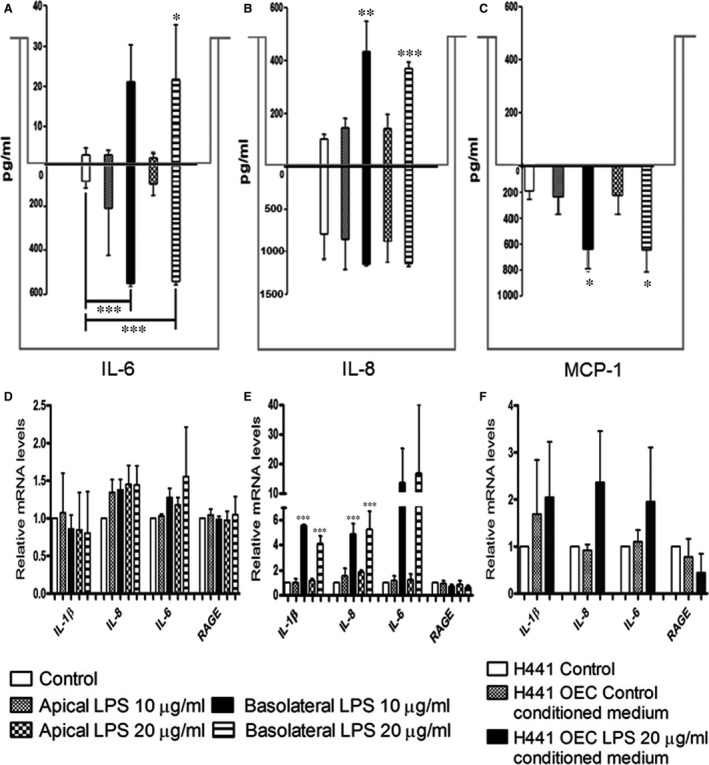
IL‐6 **(A)**, IL‐8 **(B)** and MCP‐1 **(C)** protein expressions in the supernatants of co‐cultures when treated with 10 or 20 μg/ml of LPS for 48 hrs were measured using an enzyme‐linked‐immunosorbent assay. In co‐cultures, supernatants were collected from the apical (values above zero) and the basolateral (values below zero) compartment of the Transwell^®^ filters after exposure to 10 or 20 μg/ml of LPS from the apical or the basolateral compartment for 48 hrs. Relative quantification of gene expression of the pro‐inflammatory factors *IL‐1*β*, IL‐6, IL‐8, RAGE* in H441 monocultures **(D)**, co‐cultures **(E)**, of H441 when treated with 10 or 20 μg/ml of LPS for 48 hrs, **(F)** H441 monocultures treated with 20 μg/ml of LPS‐treated OEC conditioned medium on day 5 of culture for 48 hrs was investigated by quantitative real‐time PCR. *RPL13A* was used as an endogenous standard to normalize the data, and control was set to 1. Each bar represents the mean + S.D. of three independent experiments (*P < 0.05, **P < 0.01 and ***P < 0.001, annova).

**Figure 7:**
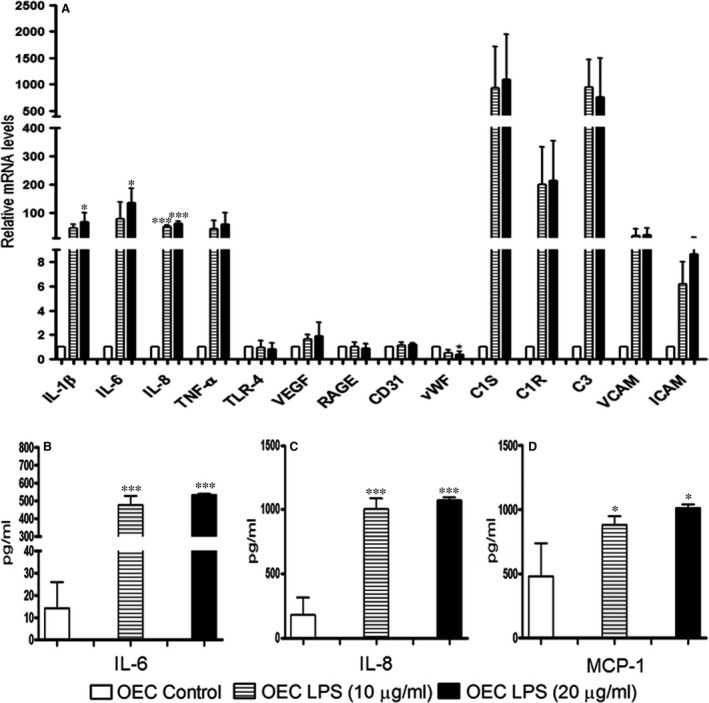
Relative quantification of gene expression of pro‐inflammatory factors, matrix, tight junctional molecules, complement pathway molecules and endothelial markers in OEC monocultures when treated with 10 or 20 μg/ml of LPS on day 5 of culture for 48 h was investigated by quantitative real‐time PCR **(A)**. *RPL13A* was used as an endogenous standard to normalize the data, and control co‐cultures were set to 1. IL‐6 **(B)**, IL‐8 **(C)** and MCP‐1 **(D)** protein expressions in the supernatants of OEC monocultures when treated with 10 or 20 μg/ml of LPS for 48 hrs were measured using an enzyme‐linked‐immunosorbent assay. In OEC, monocultures cells were cultured on 24‐well plates, and supernatants were collected after exposure to 10 or 20 μg/ml of LPS. Each bar represents the mean + S.D. of three independent experiments (*P < 0.05, and ***P < 0.001, annova).

LPS is known to induce inflammatory cytokines interfering with barrier properties and barrier function, nevertheless cell and site‐specific response in the alveolar‐capillary barrier might differ, and the origin of cytokines is often difficult to refer to individual cell types or barrier compartments. We evaluated the concentrations of inflammatory cytokines (IL‐6, IL‐8, MCP‐1) in the individual compartments of the co‐culture after exposure to LPS either from the apical or basolateral side using ELISA (Fig. 6A–C). In addition, gene expression of inflammatory cytokines was evaluated in H441 cells in response to LPS in mono‐ and in co‐cultures (Fig. 6D‐F). Furthermore, we investigated the inflammatory activation of endothelial cells in monocultures by ELISA and real‐time PCR as described in the following sections and Figure 7.

In the ELISA experiments using cell culture supernatants, the pro‐inflammatory cytokines such as IL‐6 (Fig. 6A) and IL‐8 (Fig. 6B) increased in the apical and basolateral medium upon treatment with LPS from the basolateral compartment of the co‐cultures. In addition, the cytokine concentrations in the basolateral compartment were much higher compared to their apical counterparts for all tested cytokines. In contrast to the findings for stimulation from the basolateral side, no significant changes were observed upon treatment from the apical compartment of the co‐culture. In addition, in the monocultures of H441, IL‐6 and IL‐8 were not detectable in ELISA experiments (data not shown). Similar to the results for IL‐6 and IL‐8, MCP‐1 expression was detected in basolateral compartment and was highly up‐regulated upon LPS treatment from the basolateral compartment in the co‐cultures (Fig. 6C). In semiquantitative real‐time PCR, we noticed an up‐regulation of IL‐1β, IL‐8, IL‐6 and a down‐regulation of RAGE in H441 cells after the basolateral LPS treatment of the co‐culture (Fig. 6E). No changes were noticed in the corresponding monocultures of H441 (Fig. 6D) or in co‐cultures after treatment from the apical compartment (Fig. 6E). In contrast, the addition of conditioned medium from OEC exposed to LPS resulted in a tentatively increased expression of IL‐1β, IL‐8 and IL‐6 and down‐regulation of RAGE (Fig. 6F).

### Gene expression in response to LPS in OEC Monocultures

Expression of a series of molecules in response to LPS was analysed in OEC monocultures including inflammatory cytokines but also endothelial cell markers or molecules used as indicators to document the inflammatory activation of endothelial cells. LPS treatment induced up‐regulated gene expression of VCAM‐1 and ICAM‐1 and also induced factors involved in the complement system such as C1s, C1R, C3, as well as the up‐regulated expression of the inflammatory cytokines which were prominent in the basolateral compartments of the co‐culture after LPS as described in the upper sections. CD31, TLR4, VEGF and RAGE remained stable and vWF expression were slightly down‐regulated (Fig. 7A). In parallel, ELISA from supernatants of OECs monocultures revealed significantly increased secretion of IL‐6 (Fig. 7B), IL‐8 and monocyte chemoattractant protein‐1 (MCP‐1) (Fig. 7C) caused by exposure to LPS (Fig. 7D).

### Proteomic analysis of supernatants from LPS‐treated OEC

To identify proteins released by OEC in response to stimulation with LPS into the conditioned media, nano‐liquid chromatography–mass spectrometry (LC‐MS) was performed using a set of cell culture supernatants from four OEC donors. To remove low molecular weight contaminants and salts from the samples and to concentrate proteins, supernatants were supplied to a SDS‐PAGE but only migrated into the stacking gel without further protein separation 19. After tryptic digestion, the resulting peptides were separated via ion‐paring reversed phase chromatography and analysed online by electrospray Orbitrap‐tandem MS, followed by database search for protein identification. The strict identification parameters applied (at least two tryptic peptides per protein, with at least one unique peptide, all peptides identified with high confidence (1% false discovery rate)) allow unambiguous identification.

Overall, we identified 728 different proteins. Different numbers of secreted proteins were identified in the conditioned media from the four donor samples with the majority of these proteins observed both in controls and LPS‐treated samples (Table S1).

Proteins identified in the supernatants of at least two more treated samples than control samples are summarized in Table 2 and include several proteins involved in endothelial activation on response to LPS and inflammation, vascular permeability control and regulation of angiogenesis, immune defence and proteolytic activity with high relevance for the ACB.

**Table 2:** Proteins identified in LPS‐treated donor OEC supernatants via LC‐MS. Four OEC supernatants were analysed for each treatment, and the number of times a specific protein was identified is listed. All proteins were identified under strict parsimony criteria (one or more unique peptides and at least two peptides in total) at an estimated FDR of 1%

Accession	Description	Identified in × out of four samples		
		Control	10 μg/μl LPS	20 μg/μl LPS
P09341	Growth‐regulated alpha protein	0	3	4
P19320	Vascular cell adhesion protein 1 (VCAM)	0	3	2
P14923	Junction plakoglobin	0	3	2
P09603	Macrophage colony‐stimulating factor 1	0	2	3
P25774	Cathepsin S	0	2	2
P00736	Complement C1r subcomponent	0	2	2
P09871	Complement C1s subcomponent	0	1	2
O15123	Angiopoietin‐2	1	3	3
P01024	Complement C3	1	3	3
P02771	Alpha‐fetoprotein	1	3	2
P19823	Inter‐alpha‐trypsin inhibitor heavy chain H2	1	3	2
P05362	Intercellular adhesion molecule 1 (ICAM)	1	2	2
P02774	Vitamin D‐binding protein	2	4	4
P04179	Superoxide dismutase [Mn], mitochondrial	2	4	4

Proteins exclusively found in the supernatants of LPS‐treated endothelial cells included the growth‐regulated alpha protein identified in three (10 μg/ml LPS), respectively, four (20 μg/ml LPS) of the donors after LPS stimulation.

In addition, vascular cell adhesion protein 1 (VCAM‐1), junction plakoglobin and macrophage colony‐stimulating factor 1 were identified in three, respectively, two of the treated samples but not in the controls. Cathepsin S and the complement C1r subcomponent were found in two of the four donors, whereas the complement subcomponent C1s was found in one or respectively two of treated samples but not in controls. Angiopoietin‐2, complement C3 alpha‐fetoprotein, interalpha trypsin inhibitor heavy chain H2, Intercellular adhesion molecule 1 (ICAM‐1) were found in the proteome analysis in three or respectively two of the treated donor samples but also in one of the control samples. Finally, vitamin D‐binding protein and mitochondrial superoxide dismutase were identified in all treated donor samples but also in two of the untreated samples.

## Discussion

The alveolar‐capillary barrier is highly susceptible to bacterial infection which causes a series of cellular and molecular events in the individual cell types and cellular compartments. This may finally result in the breakdown of the alveolar‐capillary barrier. The present study is analysing effects of LPS on individual cell types of the alveolar‐capillary barrier but also takes into account the crosstalk of epithelial and endothelial cells as a functional unit. In this study, we have shown that soluble factors secreted by endothelial cells in response to LPS treatment induce hyper‐permeability of the blood–air barrier and the immunological activation of epithelial cells, whereas the stimulation of H441 with LPS alone failed to show effects on the barrier and the immunological activation of epithelial cells. In addition, these effects are restricted to the treatment with LPS from the basolateral/endothelial compartment in the Transwell co‐culture system in accordance with the severe effects by LPS on the alveolar‐capillary barrier of the lung *in vivo* as a result of systemic infections.

Several findings in this study are suggesting a high impact of soluble factors derived from endothelial cells exposed to LPS on epithelial barrier properties. First of all, a reduction of TEER values as indicator for functional epithelial barriers was only observed in co‐cultures treated from the basolateral/endothelial compartment. These findings were further confirmed by the assessment of the paracellular permeability showing an increase in the transport rates of the test substances in the co‐culture in response to basolateral LPS exposure. Similar results in terms of epithelial cell barrier breakdown were also confirmed in experiments with conditioned medium derived from LPS‐treated OEC, thus suggesting that these effects on the epithelial barrier can be attributed to soluble factors produced by the endothelial cells.

According to the results from immunofluorescent staining, semiquantitative the real‐time PCR and Western blot analysis for the tight junctional molecules (zo‐1 and occludin) these effects on epithelial cell barrier were not caused by changes in the expression of tight junctional molecules. No significant changes in the expression of Zo‐1, occludin‐1, E‐cadherin or ß‐catenin were detected in real‐time PCR in the groups with reduced barrier properties. These results are in accordance with several reports from the literature which show that the barrier permeability in epithelial cells in response to inflammation or infection is not associated with differences in gene‐ or protein expression of tight and junctional molecules but rather controlled by modification of the corresponding junctional molecules for instance by phosphorylation and dislocation from the cellular contacts 24, 25, 26, 27. These regulation processes based on the modification of junctional proteins would allow a fast adaptation of the epithelial barrier to physiological changes. Similarly, we observed in our study a more dislocated pattern of the tight junctional molecule zo‐1 in epithelial cells with reduced barrier properties. Assessment of surface to overall protein ratios for the transmembrane tight junctional molecule occludin suggests only a tentative decrease of occludin on the epithelial cell surface in response to mediators released by endothelial cells being exposed to LPS. In this context, further experiments based on newly identified or more specified factors released by the endothelium might enable a more mechanistic insight responsible for the barrier break down and crosstalk of the compartments or cell types in the ACB.

Both endothelial cells and epithelial cells in our study showed distinct reactions towards the LPS exposure. Endothelial cells reacted towards the LPS with the abundant expression and secretion of inflammatory cytokines including high levels of IL‐1β, IL‐6, IL‐8 and TNF‐α, which is in accordance with previous reports 28 and reports concerning the role of inflammatory cytokines in the barrier break down 29, 30. In this context, the direct treatment of the co‐culture with IL‐6 from the basolateral compartment led to the decrease of the barrier (data not shown). In addition, IL‐6 is known to modulate the barrier properties of epithelial cells 31 and is widely accepted as a key molecule in acute lung injury 32, 33.

When comparing the concentrations of cytokines in the individual compartments of the co‐cultures or from the corresponding monocultures of epithelial or endothelial cells, epithelial cells secreted considerable lower amounts of inflammatory cytokines. Nevertheless, when exposed to soluble factors from LPS‐treated endothelial cells, epithelial cells in turn reacted by an up‐regulation of SP‐A, whereas SP‐C and SP‐D were significantly down‐regulated on the gene expression level. This was shown by data from co‐cultures but also verified by the data sets using conditioned medium from endothelial cells. Thus, we assume that the up‐regulation of SP‐A and the down‐regulation of SP‐C and SP‐D in epithelial cells may result from paracrine factors such as the inflammatory cytokines secreted by the endothelial cells rather than from a direct effect of LPS in epithelial cells. These observations also suggest communication mechanisms mediated by paracrine factors between these constitutional cell types in response to infection. There are several reports regarding the role of the surfactant proteins SP‐A, SP‐C and SP‐D for the alveolar defence in response to LPS. Besides the well characterized role for the collectins SP‐A and SP‐D 34, 35, 36 in LPS defence also surfactant protein C has been shown to interact directly with LPS thus also being involved in the antibacterial defence 37, 38.

In addition to the increase of inflammatory cytokines, the activation of endothelial cells by LPS was also indicated by the abundant expression of molecules such as ICAM‐1 and VCAM‐1 which are known to mediate the immune response in endothelial cells in response to bacterial infections 28. Beyond these classical indicators for endothelial activation additional key factors involved in the complement system such as C1r, C1s and C3 were strongly induced in endothelial cells by LPS in accordance with the current understanding on the role of these complement factors for the host defence in the lung 39 and for endothelial cell damage in acute lung injury and sepsis 40.

The gene expression data indicating a strong up‐regulation for the molecules VCAM‐1, ICAM‐1 C3, C1s, C1r in response to LPS were also reflected by the proteome data from the cell culture supernatants in which these molecules seem to be highly abundant and thus in principle allow their detection by LC‐MS. From the proteomic data analysis also a series of additional molecules such as growth‐regulated alpha protein, junction plakoglobin, macrophage colony‐stimulating factor 1, cathepsin S, angiopoietin‐2 (Ang‐2), Alpha‐fetoprotein, Inter‐alpha‐trypsin inhibitor heavy chain H2, vitamin D‐binding protein and superoxide dismutase (SoD) evolved as potential candidate molecules for LPS‐mediated effects also in accordance with previous reports from the literature. Some of these molecules have been reported to be effector molecules for the LPS response in endothelial cells including for instance Ang‐2, SOD 41 or are considered to be associated with cell damage in the lung to counteract elevated levels of free actin such as vitamin D‐binding protein 42, 43. Angiopoietin‐2 has been shown to be a key molecule produced mainly by the endothelium itself which induces vessel destabilization and vascular hyper‐permeability during sepsis and mediates LPS‐induced vascularization processes 44, 45. In addition, high levels of angiopoietin‐2 have been discussed as biomarker in human plasma samples from patients with an indirect ARDS 46.

For cathepsin S, multiple functions have been reported which could be associated with a barrier breakdown of the blood air barrier. Cathepsin S is a cysteine protease with elastolytic activity thus having a direct effect on elastin as an important extracellular matrix component in the alveolar epithelium 47. In addition, cathepsin S has been reported to cleave surfactant protein A 48 thus potentially influencing the immune response of epithelial cells. Furthermore, cathepsin S seems to be involved in highly important processes controlling the barrier properties including the proteolytic activation of epithelial sodium channels 49 and also has been shown to cleave several tight junctional molecules *in vitro* 50. In accordance with these different molecular actions of cathepsin S several reports emphasize its role in lung disease 51, 52.

Although we present here data from a preliminary proteomics approach we were able to identify a number of proteins in the supernatants of microvascular cells involved in LPS response in endothelial cells or which could serve as molecules of interests in LPS‐mediated lung injury in future studies. The fact that not all proteins were identified in all donor samples is certainly reflecting the natural biological variability of the four individuals. However, it must be noted that although these proteins were not always observed they may simply have been below the limit of detection for analysis or were masked by other more abundant protein species. It has to be noted that detection of molecules of low abundance, for example cytokines, by means of LC‐MS methods can barely be achieved.

## Conclusions

Epithelial barrier dysfunction and immunological activation of epithelial cells result at least partly from soluble factors and pro‐inflammatory cytokines secreted by endothelial cells under LPS treatment. More detailed studies, for example involving quantitative proteomics analysis will be an important tool for future studies to reveal the mechanisms and mediators in barrier regulation and underlying cellular crosstalk at the molecular level. On the other hand, the use of this co‐culture approach enables to analyse cell type‐specific reactions. Ongoing experiments are currently applied to assess the effects of specific compounds to stabilize the alveolar‐capillary barrier of the lung.

## Authors’ contributions

HJ, LC, FW, performed experiments, analysed and interpreted data. DS, SÖ, MK, AS revised the manuscript from a clinical perspective, AT, SF designed the study, evaluated the data and have written the manuscript. All authors read and approved the manuscript.

## Conflict of interest

Authors declare that no competing interests exist.

## Supporting information


**Figure S1.** Cellular viability based on MTS assessment in response to LPS. Values are depicted in % in relation to control for indicated treatment and cell types.

**Table S1.** Supplemental Proteomics Data set
