# Supplementary figures and images for: Site‐specific and endothelial‐mediated dysfunction of the alveolar‐capillary barrier in response to lipopolysaccharides
Source: J Cell Mol Med. 2017 Dec 5;22(2):982–98. doi: 10.1111/jcmm.13421 (PMC5783864; doi:10.1111/jcmm.13421)

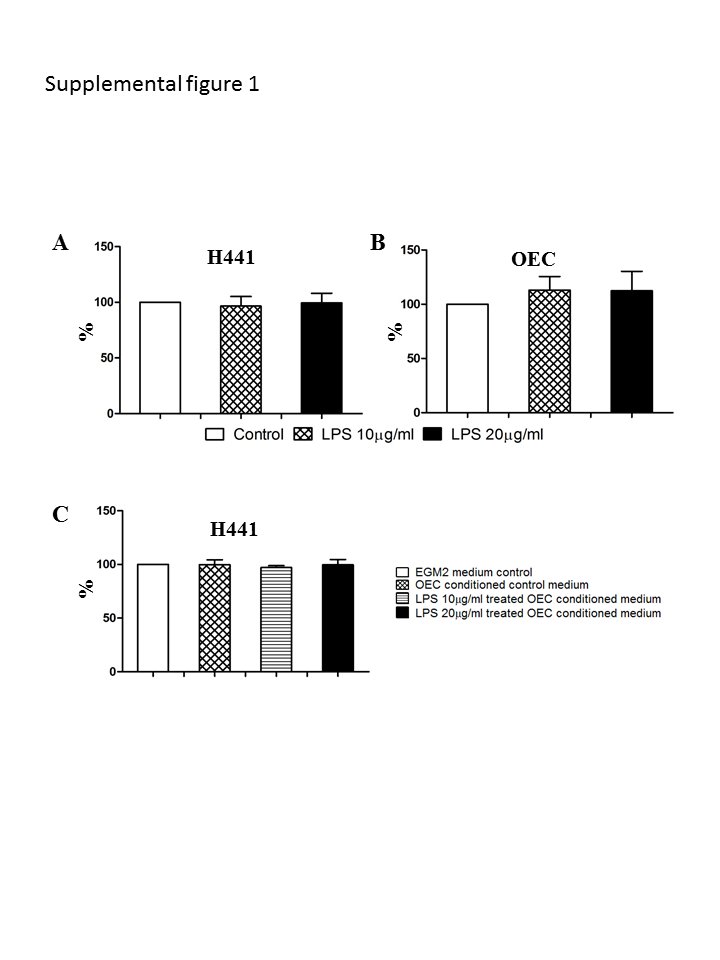

Supplement: Supplementary file 1 — Figure S1. Cellular viability based on MTS assessment in response to LPS. Values are depicted in % in relation to control for indicated treatment and cell types. [file JCMM-22-982-s001.tif]
